# Investigating risk factor and consequence accounts of executive functioning impairments in psychopathology: an 8-year study of at-risk individuals in Brazil

**DOI:** 10.1017/S0033291725100639

**Published:** 2025-07-14

**Authors:** René Freichel, Sacha Epskamp, Peter J. de Jong, Janna Cousijn, Ingmar Franken, Giovanni A. Salum, Pedro Mario Pan, Ilya M. Veer, Reinout W. Wiers

**Affiliations:** 1 Addiction Development and Psychopathology (ADAPT)-lab, Department of Psychology, University of Amsterdam, Amsterdam, The Netherlands; 2 Department of Psychology, National University of Singapore, Singapore; 3 Department of Clinical Psychology and Experimental Psychopathology, University of Groningen, Groningen, The Netherlands; 4 Center for Substance use and Addiction Research (CESAR), Department of Psychology, Education & Child Studies, Erasmus University Rotterdam, Rotterdam, The Netherlands; 5 Child Mind Institute, New York, United States; 6 National Institute of Developmental Psychiatry for Children and Adolescents (INCT-CNPq), São Paulo, Brazil; 7 Universidade Federal do Rio Grande do Sul, Hospital de Clinicas de Porto Alegre, Porto Alegre, Brazil; 8 Universidade Federal de São Paulo, Department of Psychiatry, Laboratory of Integrative Neuroscience (LiNC), São Paulo, Brazil; 9 Center for Urban Mental Health, University of Amsterdam, Amsterdam, The Netherlands

**Keywords:** executive functioning, adolescence, psychopathology, dynamic modeling

## Abstract

**Background:**

Executive functioning (EF) impairments are widely known to represent transdiagnostic risk factors of psychopathology. However, a recent alternative account has been proposed, according to which EF impairments emerge as consequences of psychopathology.

**Methods:**

Using a longitudinal cross-lagged panel network analysis approach, we tested these competing theoretical accounts at different stages during adolescence. We used data from the Brazilian High-Risk Cohort Study for the Development of Childhood Psychiatric Disorders, in which 61% of individuals at wave 1 were selected due to their high risk for psychopathology. Participants were assessed across three assessment waves during early (wave 1: *n* = 1,992, mean age = 10.20 years) and middle adolescence (wave 2: *n* = 1,633, mean age = 13.48 years; wave 3: *n* = 1,439, mean age = 18.20 years). We examined associations between working memory, inhibitory control, and broad-band measures of psychopathology.

**Results:**

During early adolescence, lower inhibitory control was a risk factor for externalizing problems that, in turn, predicted lower working memory capacity. During middle adolescence, bidirectional associations became more prominent: inhibitory control and working memory functioned as both risk factors and consequences. Externalizing problems both predicted and were predicted by poor inhibitory control. Internalizing and externalizing symptoms showed bidirectional associations over time. Externalizing problems predicted more internalizing symptoms, whereas internalizing symptoms predicted fewer externalizing problems during middle adolescence.

**Conclusions:**

Our results corroborate dynamic theories that describe executive dysfunctions as precursors and consequences of psychopathology in middle adolescence.

## Introduction

Adolescence represents a critical period marked by neurobehavioral changes, brain maturation, and cognitive-emotional development (Dahl, Allen, Wilbrecht, & Suleiman, 2018). As widely noted, this period of sensitivity may set the stage for developing adolescent mental health problems that often continue into adulthood (Lee et al., 2014; Paus, Keshavan, & Giedd, 2008). Three-fourths of all lifetime cases of mental disorders have an onset before the age of 24 years (Kessler et al. (2005), hence, onset in childhood, adolescence, or emerging adulthood. Different cognitive functions, in particular inhibitory control and working memory, have been proposed to play dual roles as being both risk factors and consequences of psychopathology in adolescence and emerging adulthood (Goschke, 2014; Huang-Pollock, Shapiro, Galloway-Long, & Weigard, 2017; Liu & Pérez-Edgar, 2019; Verdejo-García, Lawrence, & Clark, 2008; White et al., 2011). However, little is known about the specificity of these roles with respect to (a) different stages during adolescence and (b) different broad-band symptom domains of psychopathology (internalizing/externalizing). Understanding age-specific cognitive antecedents of developmental psychopathology is crucial for developing time-sensitive interventions and prevention efforts.

Executive functions are defined as a broad class of cognitive control components (Miyake et al., 2000) that include inhibitory control, shifting attention, and working memory. Different developmental profiles with a general improvement of these functions across adolescence and young adulthood have been described (e.g., Ferguson, Brunsdon, & Bradford, 2021). Executive functions rapidly develop in mid-adolescence (ages 10–15) before stabilizing in early adulthood (Tervo-Clemmens et al., 2023). These developments are accompanied by neural changes in various brain regions, including the prefrontal cortex, as well as hormonal shifts for heightened social and affective processing (Crone & Dahl, 2012). Some evidence points to variations in trajectories of different executive functions (Steinberg et al., 2008) – with linear decreases in impulsivity from age 10 but a non-linear change in sensation-seeking (increasing until age 10 before stabilizing).

Cognitive processes and their development have been more broadly associated with mental health (see RDoC framework, Insel et al., 2010). Recent studies proposed that global deficits in EF increase the transdiagnostic vulnerability to psychopathology during adolescence for at-risk individuals, such as children who experienced neglect (Schäfer et al., 2023; Wade, Zeanah, Fox, & Nelson, 2020). Similarly, global executive functions prospectively predicted the general psychopathology factor (‘p factor’; Caspi et al., 2014) during early (i.e., 10–14 years) adolescence (Martel et al., 2017; Romer & Pizzagalli, 2021). In addition to these associations with overall psychopathology, some evidence points to a potential specificity between different executive functions and broad problem domains of psychopathology (internalizing, externalizing). For instance, working memory constitutes one of the most central executive functions (Kane & Engle, 2002) and has been specifically associated with externalizing disorders in children (Huang-Pollock et al., 2017). Inhibitory control, described as the ability to control impulses or automatic behaviors, has been established as a core aspect of emotion regulation and was implicated in both externalizing (Berger & Buttelmann, 2022) and internalizing problems (Sætren, Augusti, & Hafstad, 2021) during childhood and adolescence.

The past two decades of research in developmental psychopathology have led to different theoretical approaches to integrate these findings. The risk factor theory proposes that EF impairments precede and increase individuals’ vulnerability to developing social, emotional, and behavioral problems later in life. According to the model by Carver, Johnson, and Timpano (2017), low inhibitory control may lead to internalizing or externalizing problems depending on the level of incentive sensitivity (low: internalizing, high: externalizing). Conceptually related, Wiers et al. (2018) suggested that relatively weak executive functions constitute a general risk factor for psychopathology and that temperament determines the primary area of problems (internalizing or externalizing). In the field of addiction, there is strong evidence in favor of a pre-existing cognitive vulnerability, particularly with respect to impulsive behaviors (e.g., Verdejo-García et al., 2008; White et al., 2011), going back to the classical work of Gorenstein and Newman (1980). Another study on executive functioning deficits in daily life (Letkiewicz et al., 2014) found that poor EF prospectively predicted depressive symptoms, while the reverse pathway was not present.

A contrasting theory, namely the ‘complication’ (Maasalo et al., 2021), ‘consequence’, or ‘scar’ account, suggests that EF impairments may represent consequences of psychopathological processes present in internalizing and externalizing disorders. Thus, weak EF may constitute a consequence of psychopathology. A recent study among 7–9-year-old children found evidence in favor of this consequence account for externalizing symptoms that constrained the development of inhibitory control (Maasalo et al., 2021). In addition, there is also some evidence in favor of a consequence account, with early substance use negatively impacting the development of executive functions in adolescence, with the strongest evidence from animal studies (e.g., Spear, 2018), although the evidence is weaker in human development (e.g., see the systematic review by de Goede et al., 2021). At the level of broad-band symptom domains, more internalizing and externalizing symptoms among 13–14 year old adolescents predicted lower EF 3–4 years later on (Brieant, King-Casas, & Kim-Spoon, 2022). A recent study using the Adolescent Brain Cognitive Development Study showed that p-factor scores, which represent a general dimension of psychopathology across internalizing and externalizing symptoms, prospectively predicted change in EF, but the reverse direction was also present (Romer & Pizzagalli, 2021). This suggests that EF may serve a dual role, acting both as a risk factor for the development of psychopathology and as a consequence of it.

Nevertheless, the majority of studies in humans explicitly focused on the ‘risk factor’ account by including different executive functions as predictors and symptom measures as outcomes (Freichel, Pfirrmann, de Jong et al., 2023). Thus, less is known about how various executive functions are longitudinally associated with internalizing and externalizing symptoms at different stages of development. Existing studies on the prospective associations between different EFs, on the one hand, and both internalizing and externalizing symptoms, on the other hand, show several characteristics that constrain the possibility to differentiate between the risk factor versus consequence account of EFs in psychopathology: (1) Most studies focused on late childhood and early adolescence, and little is known about associations during middle adolescence; (2) through the use of traditional statistical approaches, executive functions have been commonly treated as sole predictors; (3) studies spanning longer time periods during adolescence typically examined average changes across time, and they thus failed to capture potential changes in the structure of associations (‘covariances’) at different change points during adolescence (Freichel, Pfirrmann, Cousjin et al., 2023).

With data from the Brazilian High-Risk Cohort Study for Mental Conditions, a large longitudinal dataset of adolescents (Salum et al., 2015) assessed during three waves, the present study aimed to fill these critical caveats by addressing two key research questions: (1) Are particular domains of EF, specifically working memory and inhibitory control, risk factors for or consequences of both internalizing and externalizing symptoms? Based on prior studies (e.g., Yang et al., 2022), we predicted that inhibitory control would be a general risk factor for both. Moreover, we predicted that working memory would be a risk factor specific to externalizing symptoms. (2) Do these associations differ between different stages of adolescence? We investigated the associations between cognitive measures and internalizing/externalizing symptoms through the use of a novel methodological approach, namely cross-lagged network analysis (Wysocki, Rhemtulla, Bork, & Cramer, 2022). This exploratory, methodological approach allowed us to study the dynamic interplay (of risk factors and consequences) between specific waves without presupposing the role of EFs as either predictors or outcomes in symptom development.

## Materials and methods

### Data source and procedure

We used data from the Brazilian High-Risk Cohort Study for the Development of Childhood Psychiatric Disorders (BHRCS). This longitudinal panel study recruited a school-based community sample across two cities in Brazil (São Paulo, Porto Alegre). The recruitment targeted both high-risk children (i.e., based on extensive family history of mental disorders screening) and a randomly selected community sample. Our analyses included the entire sample, which combines both high-risk and randomly selected participants, to ensure sufficient variability in all symptom and cognitive measures. This approach was essential as network analyses require variability to capture patterns of covariance between different levels of cognitive functioning and symptom development. Participants were invited to three study visits (wave 1: years 2010–2011, wave 2: years 2013–2014, wave 3: years 2017–2019) that included the administration of clinical interviews and neuropsychological assessments. The study design and sample selection are described in more detail elsewhere (Salum et al., 2015). The study was approved by the ethical committees of the universities at each site, and all parents of participants provided informed consent.

## Measures

### Psychopathology

**CBCL.** The Child Behavior Checklist (CBCL, Brazilian version) (Bordin, Mari, & Caeiro, 1995) was used as a parent-report questionnaire to assess psychopathology symptoms. A parent or caregiver completed the checklist with 120 items that assessed emotional-behavioral problems (Bordin et al., 2013), specifically the syndrome scales: anxious/depressed, withdrawn, somatic complaints, social problems, thought problems, attention problems, rule-breaking problems, aggressive behavior, and other problems. These dimensions can be grouped into two broad-band scales (Cohen, Gotlieb, Kershner, & Wehrspann, 1985): internalizing symptoms (withdrawal, somatic complaints, anxiety/depression) and externalizing symptoms (rule-breaking problems, aggressive behavior). The CBCL was shown to be a valid assessment tool across cultures (Ivanova et al., 2007).

**ABCL.** At the third measurement wave, participants aged 18 and above were administered the Adult Behavioral Checklist (ABCL, Achenbach & Rescorla, 2003). The ABCL is part of the Achenbach System of Empirically Based Assessment and consists of 118 items that are completed by a close informant (e.g., partner, parents, friends). Similar to the CBCL (for participants below 18 years of age), we derived two broad-band scales (internalizing: withdrawn, somatic complaints, anxious/depressed; externalizing: rule-breaking problems, aggressive behavior) as well as separate empirically derived scales.

### Executive functions

**Digit span task.** A digit span task (forward/backward) from the Wechsler Intelligence Scale for Children (Wechsler, 2002) was used to assess short-term working memory capacity in the context of verbal information. Participants were instructed to listen to a sequence of numbers and repeat it either forward or backward. As the sequence got increasingly longer, the task became more difficult.

**Corsi blocks.** A corsi blocks (forward/backward) task (Vandierendonck, Kemps, Fastame, & Szmalec, 2004) assessed the short-term working memory capacity of visual–spatial information. Participants were instructed to repeat a spatial sequence a researcher indicated by tapping up to nine identical blocks. We first standardized the backward digit span scores from both tasks (digit span and corsi block span) and then averaged these scores for every participant to create one aggregate measure of working memory. This was considered appropriate as both tasks’ scores correlated moderately (*r* = 0.46, *p* < 0.01). This process also increased the reliability of the measure and allowed us to prevent issues of multicollinearity (i.e., two strongly interconnected nodes) that are common problems in network analysis (Borsboom et al., 2021). Higher averaged digit span scores indicate better working memory capacity.

### Inhibitory control

A Go/no-go task (Bitsakou, Psychogiou, Thompson, & Sonuga-Barke, 2008) was used to assess inhibitory control. Participants were instructed to press buttons indicating the direction of arrows as soon and as accurately as possible. When a double-headed arrow appeared, participants were instructed to stop pressing the button (no-go). There were 75 go-trials and 25 no-go trials. All stimuli were displayed for 100 ms, and the intertrial period was 1500 ms. As relevant measures of inhibitory control, we included participants’ average RT of correct Go trials and the number of commission errors. The use of these two distinct measures was considered appropriate as they capture different dimensions of inhibitory control and may account for a possible speed-accuracy tradeoff. We used observed scores for the cognitive task measures, rather than latent variables, to avoid issues related to disattenuation that can arise when measurement error is removed from only some variables in the model. We removed outlier reaction times and accuracy scores with an absolute z-score equal to or larger than 4 (Brunnekreef et al., 2007). To facilitate interpretation, we inverted the two indices of inhibitory control (reaction times and error) such that higher scores indicate better inhibitory control (lower reaction times and higher accuracy).

### Data preprocessing

For all analyses, we only included individuals with no missing data at the first wave for the CBCL measures and cognitive tasks (digit span, corsi-blocks, Go/NoGo task). In the Supplementary Table S1, we provide the proportion of missingness for all individual subscales/measures. The second analysis (cross-lagged network analysis from wave 2 to wave 3) included individuals with available CBCL (age below 18 years) and ABCL (age above 18 years) at the third wave. The distributions for all subscales of the available CBCL and ABCL measures appeared similar, and thus, we first standardized the CBCL/ABCL subscales separately before integrating them into the model.

### Cross-lagged network analysis

To examine the temporal associations between cognitive markers and symptoms, we used cross-lagged network analysis (Wysocki et al., 2022). This approach, as used in extant literature (Freichel, Pfirrmann, de Jong et al., 2023; Zainal & Newman, 2022b), implements a series of regularized regressions to estimate cross-lagged (different nodes predicting each other over time) and autoregressive (node predicting itself over time) effects at every change point. The Least Absolute Shrinkage and Selection Operator (LASSO) with 10-fold cross-validation (to find the optimal γ-value) was used to shrink weak estimates to zero. The cross-lagged estimates describe temporal associations between nodes while controlling for all other variables in the network. This cross-lagged network analysis method has two key benefits that are important in the present application: (1) The model identifies temporal associations without requiring an a-priori specification of predictors and outcomes that are based on theoretical assumptions about the nature of EF in symptom development; (2) By controlling for all other variables in the network, the model can inform conclusions about the relative importance of different measures, such as executive functions. Considering the variance in the age range of our sample (within every wave), we included age as a covariate in the model following the approach of Funkhouser et al. (2021) and Zainal and Newman (2022a). This meant that age was an additional predictor (covariate); however, it was not predicted by any other variable in the model. Similarly, we conducted a sensitivity analysis including gender as a covariate. The models were estimated using the *glmnet* (Friedman et al., 2017) and visualized using the *qgraph* (Epskamp et al., 2012) R packages. We used non-parametric bootstrapping procedures (Epskamp, Borsboom, & Fried, 2018) with 1,000 bootstraps to examine the accuracy of the estimated edge weights.

## Results

### Sample characteristics

Table 1 summarizes demographic and clinical characteristics at all three waves separately for the high-risk and randomly selected population samples. The high-risk group comprised 60.89% of the sample. Boys were slightly overrepresented overall, but there were no significant differences in gender distribution between samples. At the aggregate level, we found notable trends with increases in internalizing symptoms (in particular withdrawn-depressed problems) across waves, while externalizing symptoms peaked during the second wave. Participants in the high-risk sample were slightly older (<0.3 years). The high-risk group exhibited significantly higher levels of psychopathology across all CBCL subscales, with internalizing and externalizing symptoms showing consistent differences between the groups at all three waves.

**Table 1.** Sample characteristics at all waves

	Wave 1		Wave 2		Wave 3		Significance
	High-risk	Randomly selected	High-risk	Randomly selected	High-risk	Randomly selected	
N	1213	779	997	636	874	565	
Sex: % female	43.94	47.5	43.33	45.75	41.6	46.18	
Age in years	10.31 (1.92)	10.02 (1.90)	13.57 (1.94)	13.32 (1.89)	18.31 (2.02)	18.03 (1.97)	w1, w2, w3
Internalizing sum score	9.53 (8.95)	7.11 (7.60)	9.98 (8.85)	7.92 (7.91)	10.76 (9.19)	9.31 (8.53)	w1, w2, w3
Externalizing sum score	9.53 (9.56)	6.91 (8.15)	9.43 (8.90)	7.42 (8.20)	8.99 (8.79)	7.12 (7.52)	w1, w2, w3
Anxiety subscale	4.49 (4.22)	3.42 (3.75)	4.25 (4.07)	3.46 (3.83)	4.46 (4.17)	3.98 (4.17)	w1, w2
Withdrawn-depressed subscale	2.24 (2.89)	1.56 (2.34)	3.03 (3.29)	2.26 (2.73)	3.53 (3.31)	3.01 (3.08)	w1, w2, w3
Somatic problems subscale	2.80 (3.35)	2.12 (2.79)	2.70 (3.07)	2.20 (2.73)	2.82 (3.30)	2.34 (3.05)	w1, w2
Thought problems subscale	2.33 (3.33)	1.62 (2.64)	1.98 (2.79)	1.55 (2.51)	2.25 (2.89)	1.55 (2.28)	w1, w2, w3
Attention problems subscale	5.45 (4.80)	3.83 (4.13)	5.08 (4.54)	4.02 (4.32)	4.46 (4.38)	3.23 (3.74)	w1, w2, w3
Rule-breaking problems subscale	2.31 (3.03)	1.73 (2.65)	2.50 (3.07)	1.93 (2.89)	2.91 (3.36)	2.27 (2.71)	w1, w2, w3
Aggressive problems subscale	7.23 (7.04)	5.18 (5.92)	6.93 (6.37)	5.48 (5.85)	6.04 (6.03)	4.82 (5.33)	w1, w2, w3
GoNoGo mean RT correct Go	450.85 (126.83)	445.69 (119.83)	347.65 (91.04)	339.98 (79.36)	305.24 (70.01)	292.52 (62.13)	w3
GoNoGo percentage commission errors	25.56 (21.78)	24.40 (22.00)	20.23 (21.33)	17.79 (20.28)	10.36 (14.05)	9.70 (12.51)	w2
WM digit span task regular	3.54 (1.60)	3.55 (1.52)	4.36 (1.78)	4.46 (1.67)	4.45 (1.92)	4.55 (1.76)	
WM task corsi	4.73 (2.15)	4.82 (2.04)	5.90 (2.14)	5.97 (2.12)	6.79 (2.37)	7.09 (2.25)	w3

*Note:* High-risk refers to the high-risk sample. Randomly selected refers to the randomly selected community sample. The range of the CBCL scales differs between syndromes. *N refers* to the number of participants with no missing data on the CBCL/ABCL internalizing and externalizing measures. w1 = wave 1, w2 = wave 2, w3 = wave 3. To facilitate data interpretation, we display only the CBLC scores and the raw scores (RT and errors) of the inhibitory control tasks. Chi-squared tests were used to compare gender distributions, while two-sided t-tests were used to assess differences in all symptom and cognitive measures. The significance columns refer to *p* < 0.05. The Supplementary Table S3 includes all relevant test statistics.

Abbreviations: *CBCL =* Child Behavior Checklist, *RT* = Reaction Time, *M* = Mean, *SD* = Standard Deviation, *ms* = milliseconds, w = wave.

Participants’ cognitive performance also steadily improved throughout the three waves, with higher speed and accuracy in both the inhibition and working memory tasks during the later waves. The high-risk and randomly selected samples showed significant differences in inhibitory control, where the randomly selected group showed fewer errors at wave 2 and faster reaction-times at wave 3. Moreover, individuals in the high-risk group exhibited lower working memory capacity at wave 3.

There was substantial attrition throughout the three waves, with 35.14% of individuals from the first wave dropping out during the study. A supplemental analysis (see Supplementary Table S2) revealed that age, gender, externalizing symptoms, and cognitive performance at wave 1 were not associated with dropout. However, higher internalizing symptoms at wave 1 predicted a lower probability of dropout.

### Associations during early adolescence

**Figure 1.**
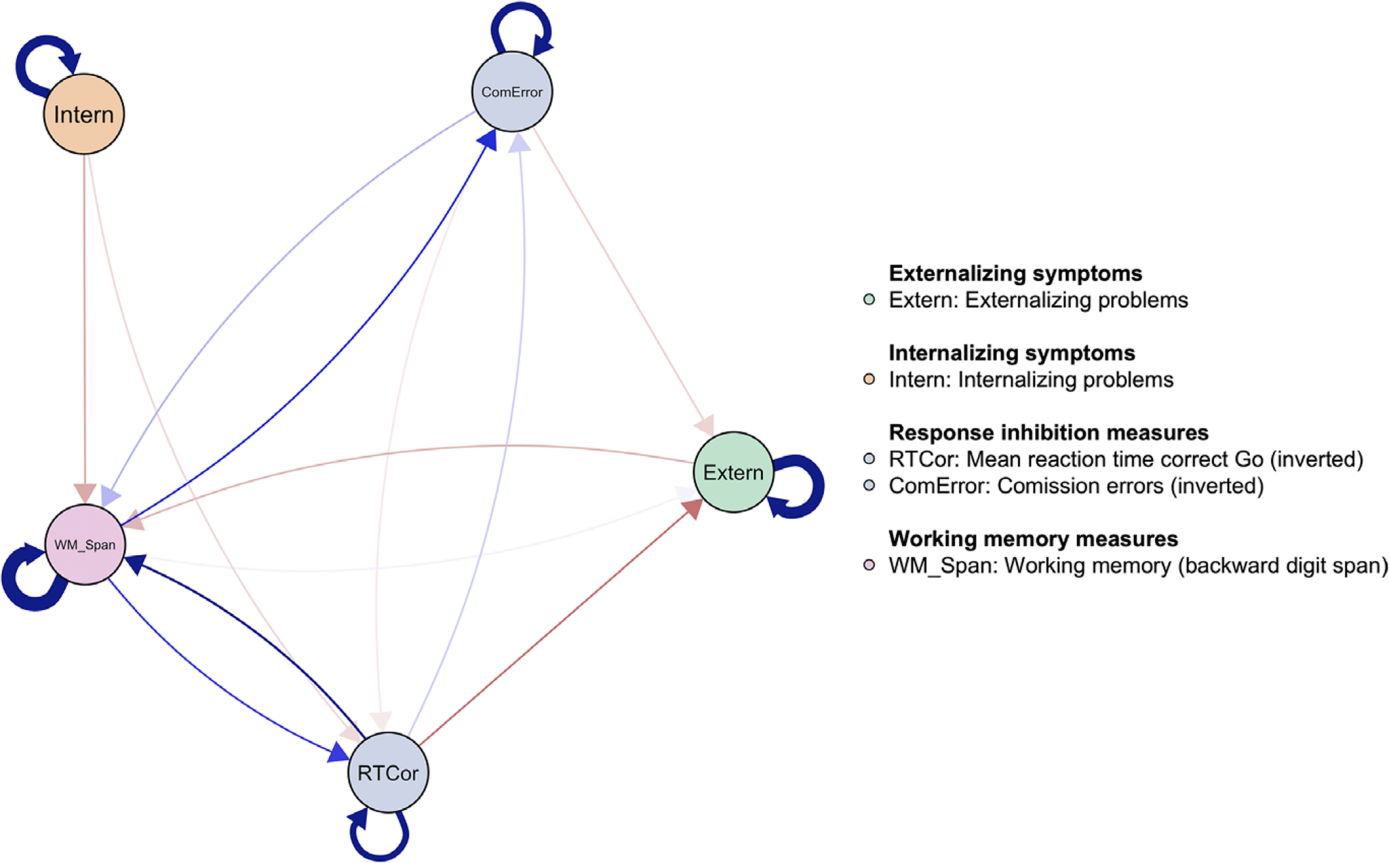
Wave 1 to wave 2 temporal associations (broad-band scales). *Note.* The network depicts temporal associations from wave 1 to wave 2. Each node represents a construct measured at both waves. Outgoing edges (arrows) reflect how a construct at wave 1 predicts another construct at wave 2, while incoming edges reflect how a construct is predicted by other constructs from wave 1. The outcome measures corresponding to the inhibitory control tasks have been inverted to facilitate interpretation. Higher scores on all cognitive control measures (RTCor, ComError, WM_Span) indicate better performance. The colors (blue = positive; red = negative) and thickness of the edges represent the direction and strength of associations, respectively. The edge weights are scaled based on the highest absolute edge weight in the network. The circular arrows on top of each node indicate autoregressive effects (i.e., the extent to which a construct predicts itself over time from wave 1 to wave 2).

Figure 1 visualizes the temporal associations occurring during early adolescence (from wave 1 to wave 2, age: *M*_wave 1_ = 10.2, *M*_wave 2_ = 13.5). Directed edges (i.e., arrows) indicate temporal associations between nodes from wave 1 predicting nodes at wave 2. At the aggregate level, both indices of inhibitory control (lower commission errors and faster reaction time) predicted fewer externalizing problems. Commission errors and the index of working memory capacity predicted each other over time. A higher working memory capacity predicted fewer commission errors, and, to a lesser extent, the opposite pathway was present. Both externalizing and internalizing problems predicted a lower working memory span over time. There was also a strong positive association between working memory capacity and speed of inhibitory control. Higher working memory capacity predicted faster reaction times in the inhibitory control task, and the reverse direction was present as well. The estimated networks were sufficiently stable (see Supplementary Figures S1and S2 for the results from our bootstrapping stability analysis).

### Predictive associations from early to middle adolescence

**Figure 2.**
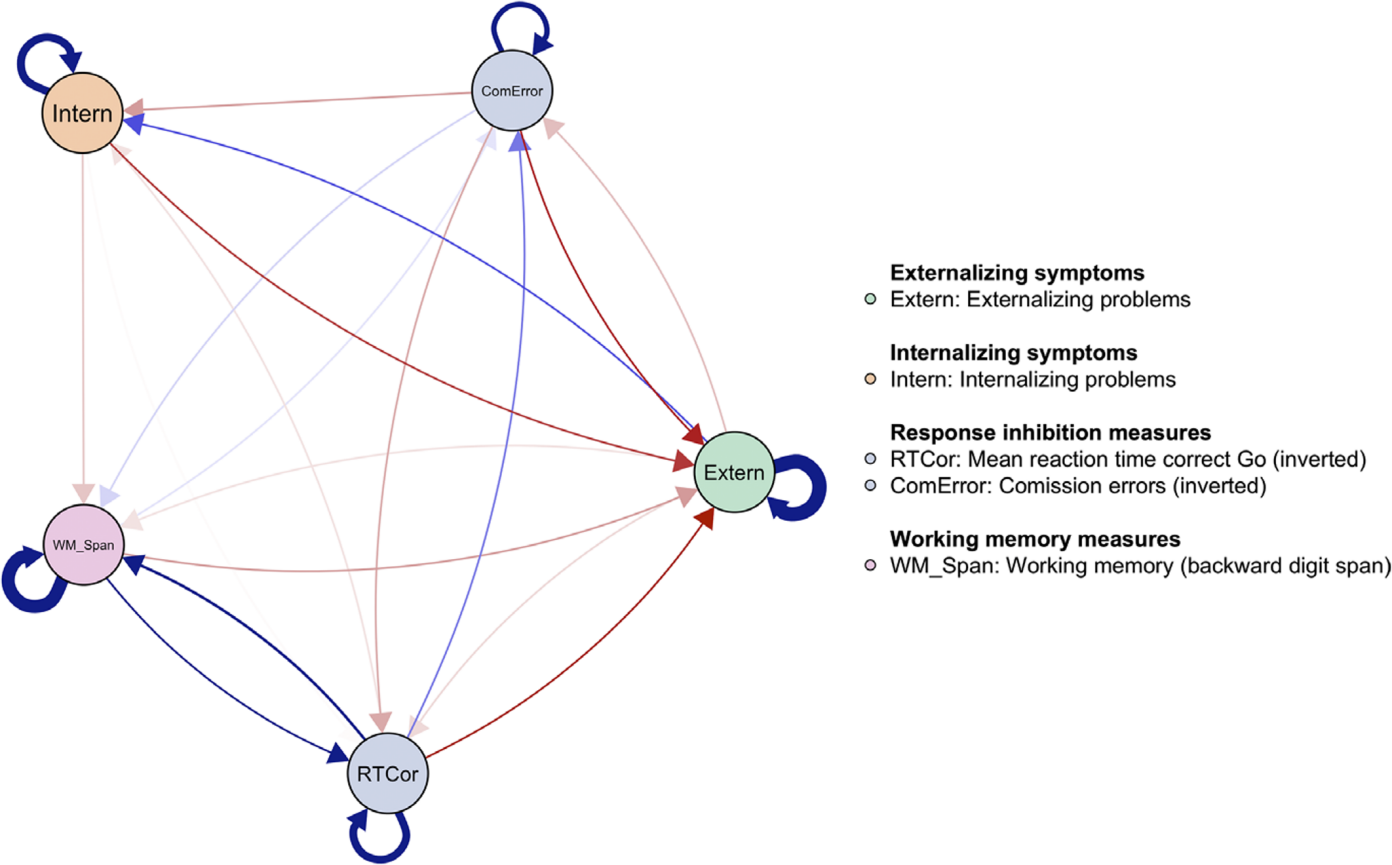
Wave 2 to wave 3 temporal associations (broad-band scales). *Note.* The network depicts temporal associations from wave 2 to wave 3. Each node represents a construct measured at both waves. Outgoing edges (arrows) reflect how a construct at wave 2 predicts another construct at wave 3, while incoming edges reflect how a construct is predicted by other constructs from wave 2. The outcome measures corresponding to the inhibitory control tasks have been inverted to facilitate interpretation. Higher scores on all cognitive control measures (RTCor, ComError, WM_Span) indicate better performance. The colors (blue = positive, red = negative) and thickness of the edges represent the direction and strength of associations, respectively. The edge weights are scaled based on the highest absolute edge weight in the network. The circular arrows on top of each node indicate autoregressive effects (i.e., the extent to which a construct predicts itself over time from wave 2 to wave 3).

Next, we examined the associations between cognitive functioning measures and symptoms during the change from early to middle adolescence (age: *M*_wave 2_ = 13.5, *M*_wave 3_ = 18.20). The aggregate temporal network (see Figure 2) is more dense (proportion of non-zero edge: 92% for waves 2–3, 72% for waves 1–2) and contains more reciprocal associations than the previous network during early adolescence. Consistent with the associations observed during early adolescence, better inhibitory control (commission errors and reaction time) predicted fewer externalizing problems. However, we also found effects in the opposite direction, with more externalizing problems predicting worse inhibitory control. There was a weak negative association between internalizing symptoms and working memory span. Moreover, better inhibitory control (fewer commission errors) predicted fewer internalizing problems. Finally, we observed reciprocal associations between externalizing and internalizing symptoms with different signs: A higher level of externalizing symptoms predicted more internalizing symptoms, whereas higher levels of internalizing symptoms at wave 2 predicted fewer externalizing problems later on (wave 3).

### Sensitivity analyses

In addition to the cross-lagged network analysis, we estimated the panel graphical vector-autoregression panel model (Epskamp, 2020) on all three waves of data to examine whether we may be able to obtain an average within-person temporal network (across the three waves). The panel GVAR model is similar to a random-intercept cross-lagged panel model, requires three waves of data, and yields partial (directed) within-person correlations. The model assumes stationarity, and given the substantial trends, we standardized the data at each time point, which inflated the model fit statistics. The panel GVAR model estimated on the detrended data showed poor fit (TLI = 0.56, CFI = 0.73, RMSEA = 0.084), further suggesting that the network structure changes over the course of the three waves. Thus, we considered a cross-lagged network analysis approach examining wave-by-wave associations defensible. Moreover, we re-estimated the cross-lagged network analyses with gender as an additional covariate. This sensitivity analysis (see Supplementary Figures S3 and S4) confirmed that key findings remain consistent when controlling for gender.

## Discussion

The present study identified dynamic associations between EF measures, namely working memory and inhibitory control, and broadband scales of internalizing and externalizing symptoms during different stages of adolescence. Our findings revealed two key pathways through which executive functions play into transdiagnostic symptom development.

### Relatively weak inhibitory control is a risk factor for externalizing symptoms throughout adolescence

During both early and middle adolescence (average ages 10–14, 14–18), relatively weak inhibitory control, indicated by high scores on commission errors and higher reaction times, predicted more externalizing symptoms. The specificity of this association has been found in prior study (Bohlin, Eninger, Brocki, & Thorell, 2012; Quach et al., 2020). Our study replicated and extended these findings because we examined a longer time frame ranging from ages 10 to 18 and controlled for a range of other variables, including working memory capacity and internalizing symptoms. Low inhibitory control may lead to emotion regulation difficulties and more impulsive behaviors. In line with this risk-account, a recent study by Hentges et al. (2020) showed that an intervention on inhibitory control during early childhood was associated with a reduction in externalizing symptoms at age 14. Further research is needed to better understand the time frame at which the negative repercussions of low inhibitory control in early adolescence can still be mitigated. Our results further indicated that externalizing symptoms predicted future internalizing symptoms during the change from early to middle adolescence. Likely, externalizing behaviors, such as delinquency or aggressive behaviors, may lead to adverse reactions from peers, with a potential loss of social status. This may include peer rejection and academic challenges – all constituting stressors that could explain the increase in internalizing symptoms later on (Weeks et al., 2016).

### Adolescent externalizing and internalizing problems predict lower working memory capacity

A novel finding from our analyses (at both change points) is that internalizing and externalizing problems predicted a lower working memory capacity over time. There may be multiple underlying mechanisms that could explain these associations. Emotional and behavioral problems may lead to extensive worrying or rumination that could tax cognitive resources, such as working memory (Levens, Muhtadie, & Gotlib, 2009). Our analyses also revealed a weak positive association between working memory capacity and externalizing problems during early adolescence (see Figure 1). Relatively high working memory capacity was associated with *higher* scores on externalizing symptoms during early adolescence, specifically with more rule-breaking behavior. We provide possible explanations for this time-sensitive role of working memory: Likely, higher working memory at age 10 allows adolescents to engage in more sophisticated, complex behaviors and social settings that may, in turn, lead to situations during which rule-breaking or externalizing behaviors can be shown. This aligns with the proposed view of social working memory as a key cognitive competency associated with individuals’ social network size (Krol, Meyer, Lieberman, & Bartz, 2018). In particular, high working memory at age 10 may put adolescents into contact with social environments that increase the likelihood of externalizing symptoms (aggressive and rule-breaking behaviors) later on. These speculations warrant further research into potential mediating factors, such as the role of peer relationships and family dynamics.

In addition to the predictive associations between EF and symptoms outlined above, we found a complex pattern of associations within the EF and internalizing/externalizing symptom domains. In both early and middle adolescence, we found that a higher working memory capacity predicted lower speed during the inhibition task and more attention problems. This is in line with previous research showing that these different cognitive functions are interrelated during development (Beattie, Schutte, & Cortesa, 2018). In addition, our findings regarding the evolving dynamic relationships among executive functions during adolescence, the distinct roles of inhibition and working memory, and the overall growth of cognitive abilities throughout align with the cognitive mutualism theory (Kievit, 2020). This theory describes that positive associations between EFs contribute to general cognitive ability during sensitive developmental windows. Future studies should further integrate these dynamic associations between different EFs in developmental psychopathology theories.

### Risk factor and consequence accounts depend on the developmental stage

**Figure 3.**
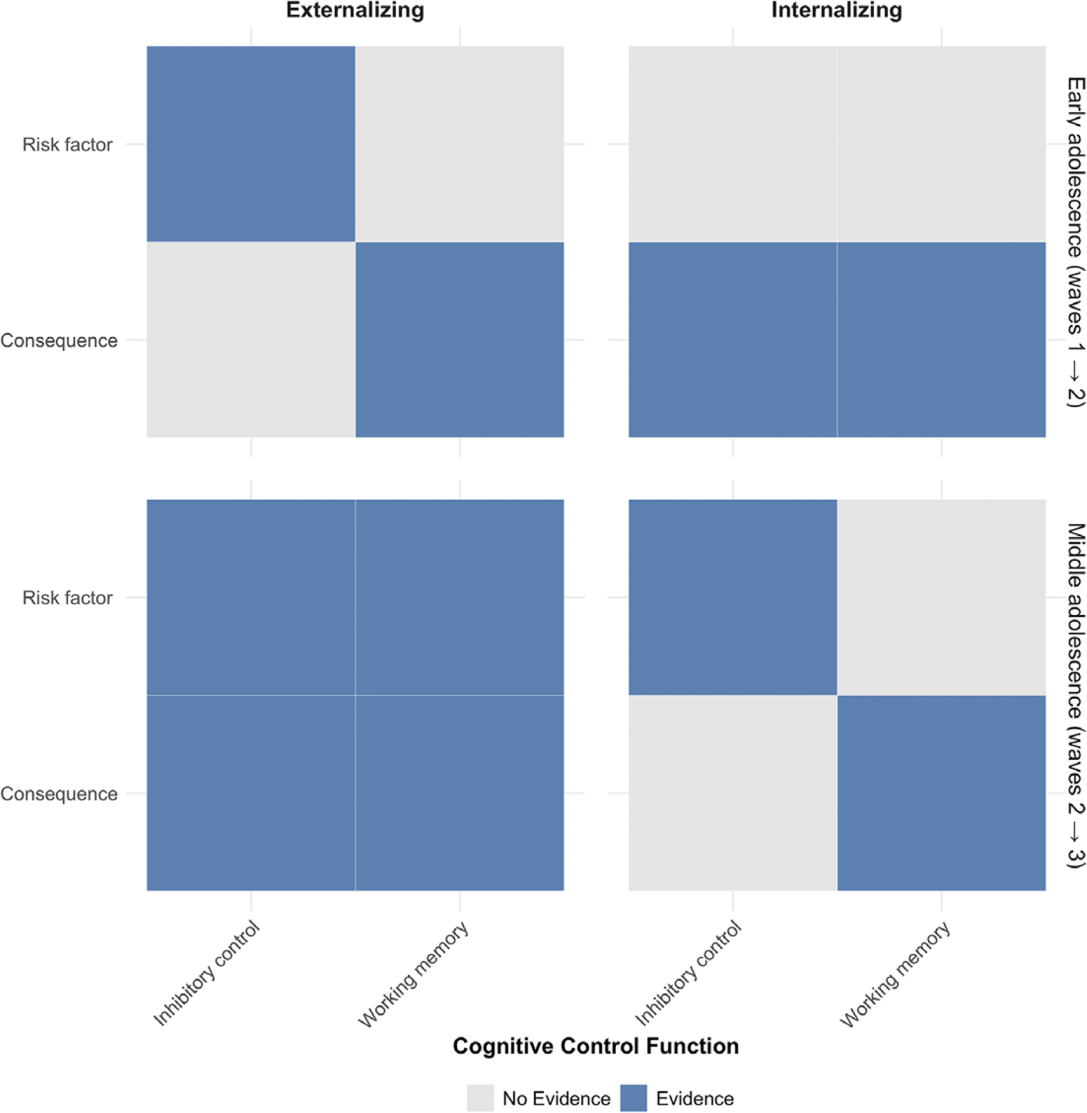
Evidence for risk factors and consequence accounts for different cognitive functions, transdiagnostic dimensions, and stages during adolescence. *Note.* This figure was based on the presence of directed edges between cognitive control functions (working memory, inhibitory control) and transdiagnostic dimensions (internalizing, externalizing symptoms) at different stages during adolescence (Figure 1: early adolescence, Figure 2: early to middle adolescence).

Altogether, our findings underscore the importance of understanding the role of executive functions in the context of different developmental stages. The conceptual Figure 3 illustrates the evidence in favor of the aforementioned theoretical accounts (risk factor versus consequence theory of cognitive dysfunction) based on our cross-lagged network models. We found evidence for the risk factor theory of inhibitory control, according to which cognitive dysfunction (see ‘C factor’, Abramovitch, Short, & Schweiger, 2021) precedes the development of externalizing symptoms in early adolescence. However, working memory in early adolescence appears to be a cognitive function that is a ‘complication’ (Maasalo et al., 2021) of this transdiagnostic symptom development. In contrast, during middle adolescence (waves 2–3), we observed more reciprocal relationships, with working memory and inhibitory control being both risk factors and consequences. Likely, psychopathology in early life may impact the normative development of executive functions (Rudd et al., 2021), which, in turn, exert their influence as catalysts of more or less adaptive developmental processes. Our study used a novel panel network analytical approach that allowed us to test different theoretical approaches by studying working memory and inhibitory control as both predictors and outcomes in parallel.

There are, however, several limitations that should be noted. First, cross-lagged panel network analysis conflates within- and between-person effects (Hamaker, Kuiper, & Grasman, 2015) and may result in a low specificity in identifying true within-person temporal effects (Freichel et al., 2024). The relevant network model for identifying within-person associations (i.e., the panel GVAR model) showed a poor model fit, even when the temporal trends were removed. This provided further evidence that the network structure indeed changes during adolescence. However, as we did not separate within- and between-person effects, these temporal estimates should not be interpreted as causal, mechanistic processes. Second, there was a significant attrition rate, in particular, during the second assessment wave, and thus, our findings may be biased. Third, we used two well-validated measures of EF, namely working memory and inhibitory control. Future studies could test temporal associations between transdiagnostic symptom measures and a wider range of executive functions, including measures of cognitive flexibility, shifting, updating, and verbal and motor speed. Deriving a common EF factor and integrating it in models of symptom dynamics may provide additional insights. Lastly, our analysis of the last assessment wave combined information from the CBCL/ABCL assessment. While this is common in studies with a wide age range (Savage et al., 2015), it is important to acknowledge that these two measures, though designed to be analogous, may assess somewhat different aspects in children versus adults. This could introduce a degree of measurement invariance that may affect the accuracy and interpretation of the observed cross-lagged effects.

A greater understanding of the dynamic associations between EF and psychopathology may provide insights into the sensitive time windows in adolescence, during which interventions may be useful. Our findings showed that during early adolescence (1) lower inhibitory control is a risk factor for externalizing symptoms and (2) working memory capacity is a consequence of externalizing symptoms. During middle adolescence, both working memory and inhibitory control serve as both risk factors and consequences of symptom development. Further research is needed to tailor cognitive developmental cascade theories to specific phases of adolescent development. The ultimate objective is to develop early intervention strategies that target relevant EFs in time to prevent an ‘escalation’ of negative symptom dynamics from arising during adolescence.

## Supporting information

Freichel et al. supplementary material Freichel et al. supplementary material

## Data Availability

The authors have obtained the data under a license, so it cannot be shared directly. .

## Supplementary material

The supplementary material for this article can be found at http://doi.org/10.1017/S0033291725100639.
