# Supplementary material for: Investigating risk factor and consequence accounts of executive functioning impairments in psychopathology: an 8-year study of at-risk individuals in Brazil
Source: Psychol Med. 2025 Jul 14;55:e192. doi: 10.1017/S0033291725100639 (PMC12315668; doi:10.1017/S0033291725100639)

**Supplementary Materials**

**Table of Content**

[Supplementary Materials Section 1: Missingness 2](#_Toc173933694)

[Supplementary Materials Section 2: Network stability 4](#_Toc173933695)

[Supplementary Materials Section 3: Sensitivity analysis including gender as a covariate 7](#_Toc173933696)

# Supplementary Materials Section 1: Missingness

**Percentage of missingness.** The percentage of missing values for each measure at the different waves is shown in Table S5.

**Table S1**

*Percentage of Missing Values for Each Measure at Waves 2 and 3*

|  | Wave 2 | Wave 3 |
| --- | --- | --- |
| Internalizing sum score | 18.02 | 27.76 |
| Externalizing sum score | 18.02 | 27.76 |
| Anxiety  subscale | 18.02 | 28.31 |
| Withdrawn-depressed subscale | 18.02 | 28.11 |
| Somatic problems subscale | 18.07 | 28.16 |
| Thought problems subscale | 18.02 | 28.11 |
| Attention problems subscale | 18.02 | 28.11 |
| Rule-breaking problems subscale | 18.02 | 28.41 |
| Aggressive problems subscale | 18.02 | 28.06 |
| Aggressive problems subscale | 18.02 | 28.06 |
| Mean RT Go | 34.29 | 42.77 |
| Percentage commmission errors | 29.47 | 35.79 |
| Digit span task | 26.56 | 34.14 |

*Note.* The percentages of missing values for each measure were calculated based on data from 1992 individuals with no missing data in wave 1. RT = Reaction Times.

**Predictors of missingness.** We investigated whether these symptom and cognitive measures at the first wave could predict dropout at any point during the study. Of all participants, 1,292 had available symptom data across all waves, while 700 individuals dropped out at some point. A logistic regression model was constructed, incorporating gender, age, internalizing and externalizing symptoms (measured at wave 1), mean reaction time in Go trials, percentage of commission errors, and digit span (all measured at wave 1) as predictors. Table S2 presents the results from the regression model.

**Table S2**

*Regression model predicting drop-out during the study using wave 1 measures*

| Predictors | Estimates |
| --- | --- |
| Intercept | 0.22 ^**^ |
| Age at wave 1 | 0.01 |
| Gender (female) | 0.01 |
| Internalizing sum score | -0.03 ^*^ |
| Externalizing sum score | 0.01 |
| GoNoGo mean RT correct Go | 0.00 |
| GoNoGo percentage comission errors | 0.01 |
| Working memory digit span | 0.00 |

*Note. * p<0.05   ** p<0.01*

**Table S3**

|  | Wave 1  t/ χ² statistic p | | Wave 2  t/ χ² statistic p | | Wave 3  t/ χ² statistic p | |
| --- | --- | --- | --- | --- | --- | --- |
| Sex: % Female | 2.279 | 0.131 | 0.83 | 0.362 | 1.245 | 0.264 |
| Age in Years | 3.277 | 0.001 | 2.618 | 0.009 | 2.652 | 0.008 |
| Internalizing sum score | 6.472 | < 0.001 | 4.888 | < 0.001 | 2.136 | 0.033 |
| Externalizing sum score | 6.542 | < 0.001 | 4.692 | < 0.001 | 2.994 | 0.003 |
| Anxiety subscale | 5.935 | < 0.001 | 3.954 | < 0.001 | 1.468 | 0.143 |
| Withdrawn-depressed subscale | 5.699 | < 0.001 | 5.1 | < 0.001 | 2.112 | 0.035 |
| Somatic problems subscale | 4.882 | < 0.001 | 3.434 | 0.001 | 1.962 | 0.050 |
| Thought problems subscale | 5.228 | < 0.001 | 3.223 | 0.001 | 3.552 | < 0.001 |
| Attention problems subscale | 8.005 | < 0.001 | 4.752 | < 0.001 | 3.978 | < 0.001 |
| Rule-breaking problems subscale | 4.502 | < 0.001 | 3.813 | < 0.001 | 2.736 | 0.006 |
| Aggressive problems subscale | 6.969 | < 0.001 | 4.702 | < 0.001 | 2.799 | 0.005 |
| GoNoGo mean RT correct Go | 0.916 | 0.360 | 1.605 | 0.109 | 3.219 | 0.001 |
| GoNoGo percentage commission errors | 1.16 | 0.246 | 2.152 | 0.032 | 0.878 | 0.380 |
| WM Digit Span Task regular | -0.169 | 0.866 | -1.056 | 0.291 | -0.994 | 0.320 |
| WM Digit Span Task corsi | -0.891 | 0.373 | -0.551 | 0.581 | -2.289 | 0.022 |

*Test statistics for comparisons* of demographic-clinical characteristics

*Note.*  This table refers to the comparisons between the high-risk and randomly selected community samples at each wave. χ² refers to the comparisons with respect to gender (male/female). Chi-squared tests were used to compare gender distributions, while two-sided t-tests were used to assess differences in all symptom and cognitive measures. The significnace

# Supplementary Materials Section 2: Network stability

**Figure S1.** *Non-parametric Bootstrapping Results for Waves 1 🡪 2 Network.
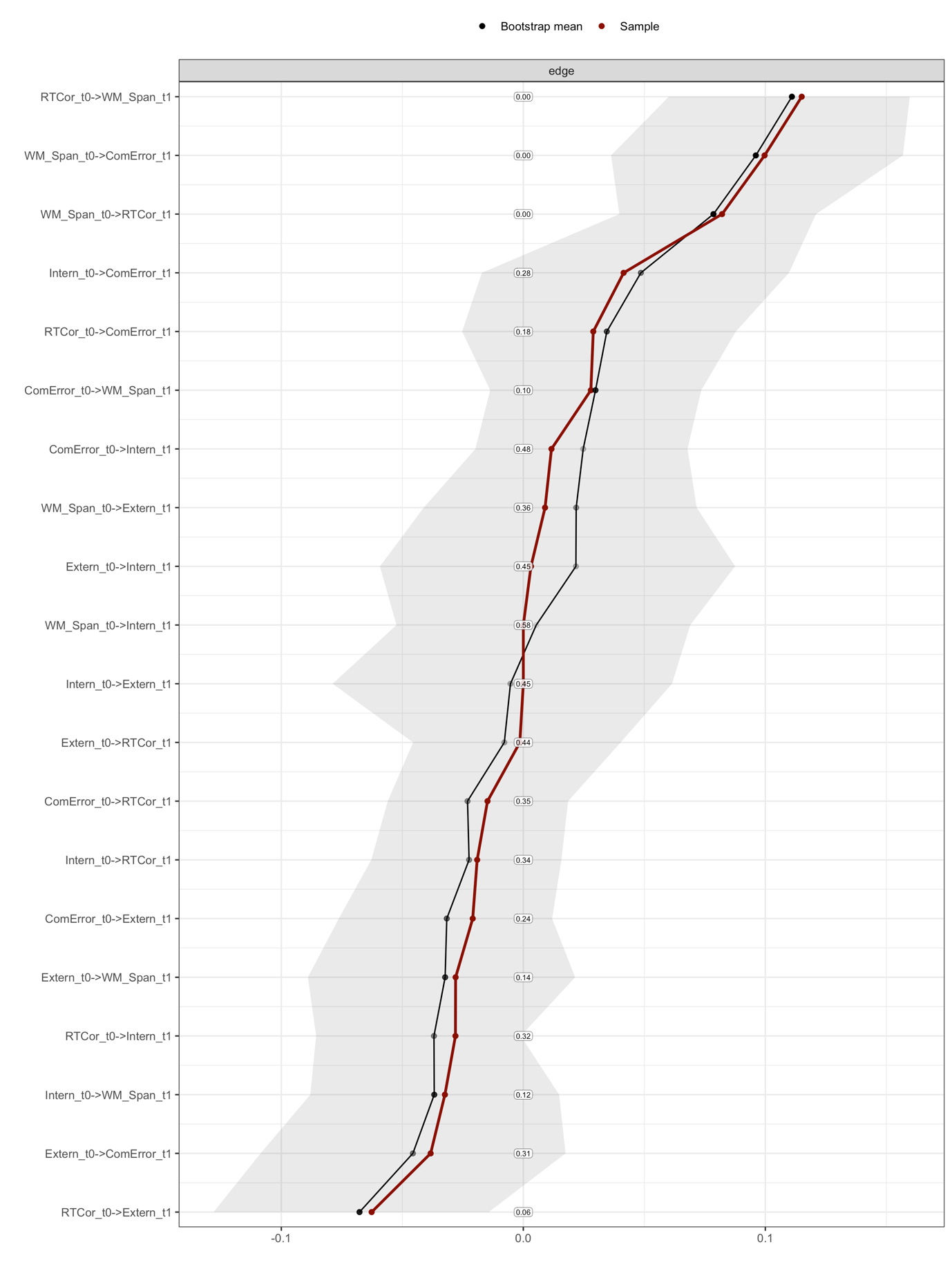
*

*Note.* t0 = wave 1, t1 = wave 2. The grey area indicates the 95% bootstrapped confidence interval of the estimated edge weights (standardized estimates) around the sampled values (in red). The middle bar denotes the proportion of estimates being zero.

**Figure S2.** *Non-parametric Bootstrapping Results for Waves 2 🡪 3 Network.*

**
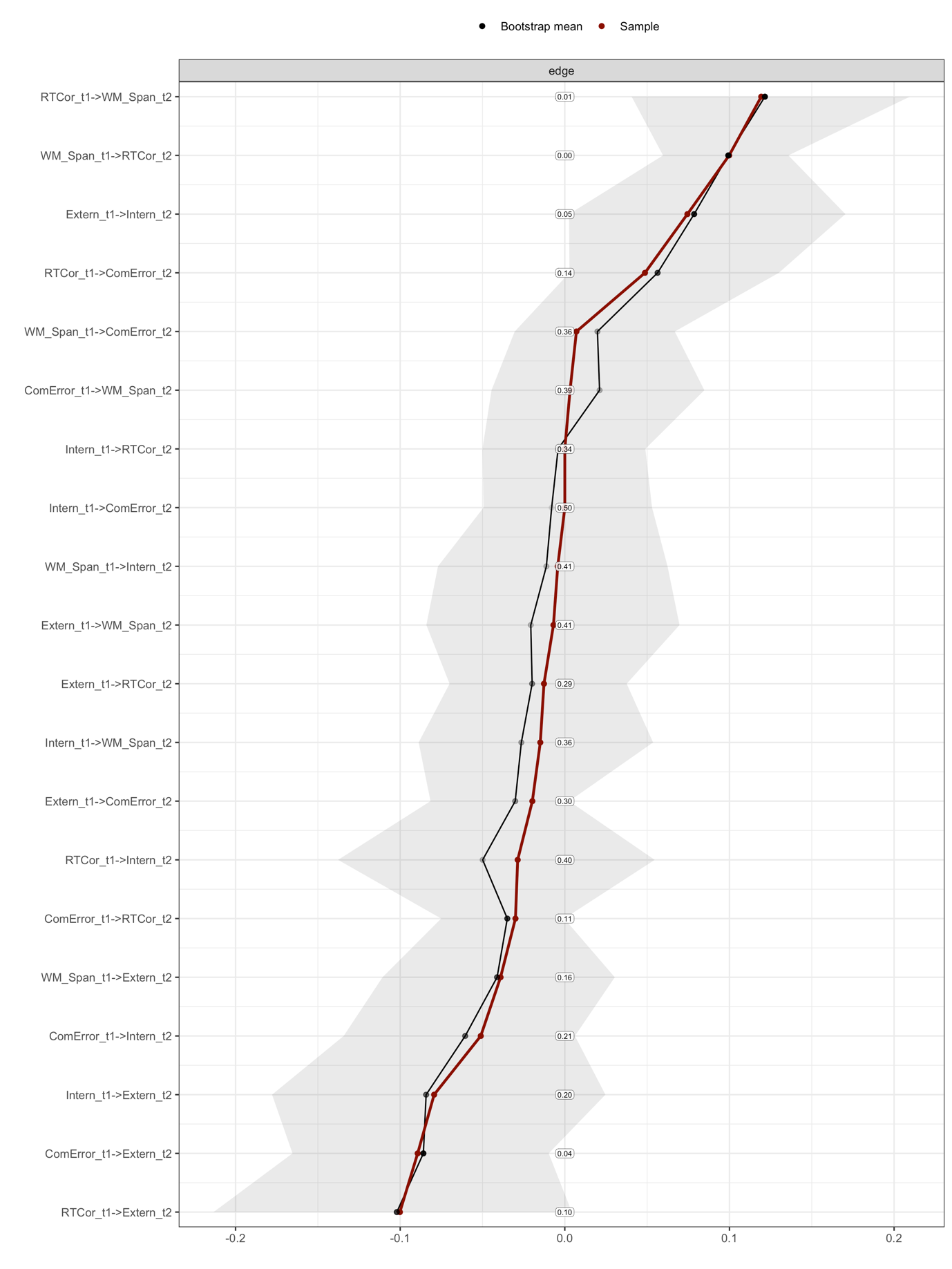
**

*Note.* t1 = wave 1, t2 = wave 2. The grey area indicates the 95% bootstrapped confidence interval of the estimated edge weights (standardized estimates) around the sampled values (in red). The middle bar denotes the proportion of estimates being zero.

# Supplementary Materials Section 3: Sensitivity analysis including gender as a covariate

**Figure S3.** *Waves 1 🡪 2 Network Including Gender as a Covariate*

**
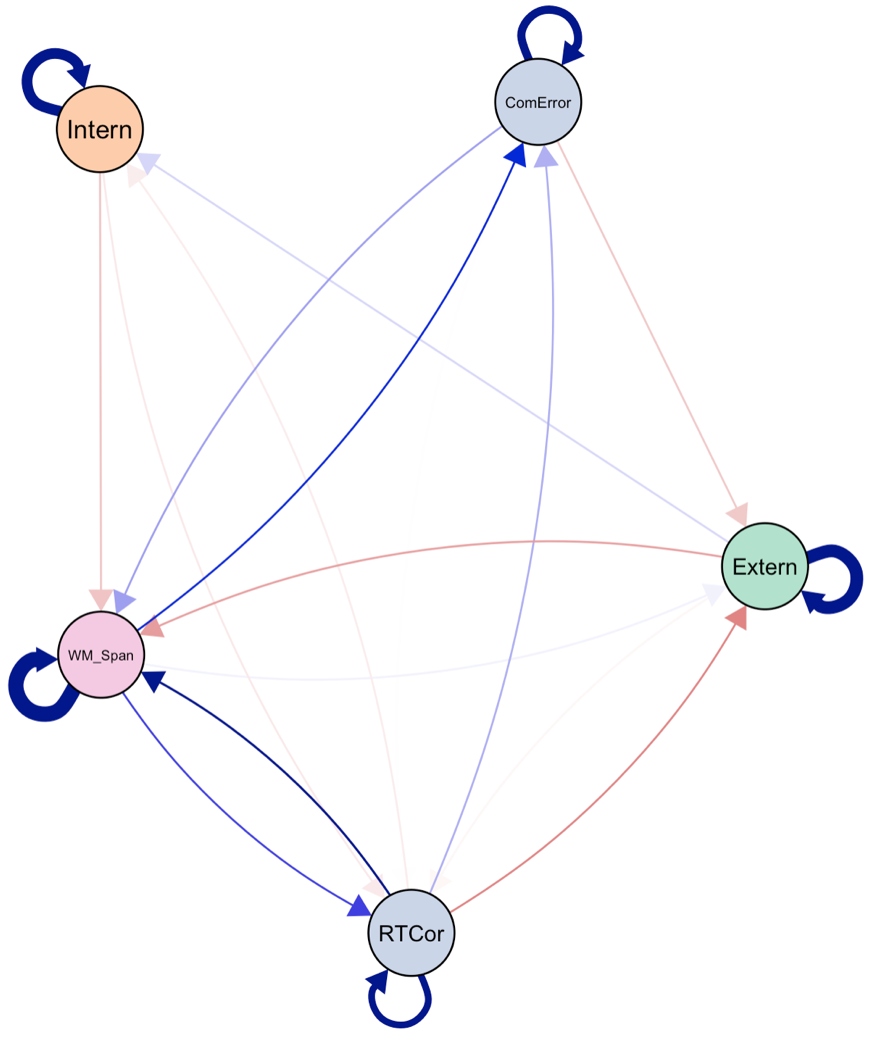
**

**Figure S4.** *Waves 2 🡪 3 Network Including Gender as a Covariate*


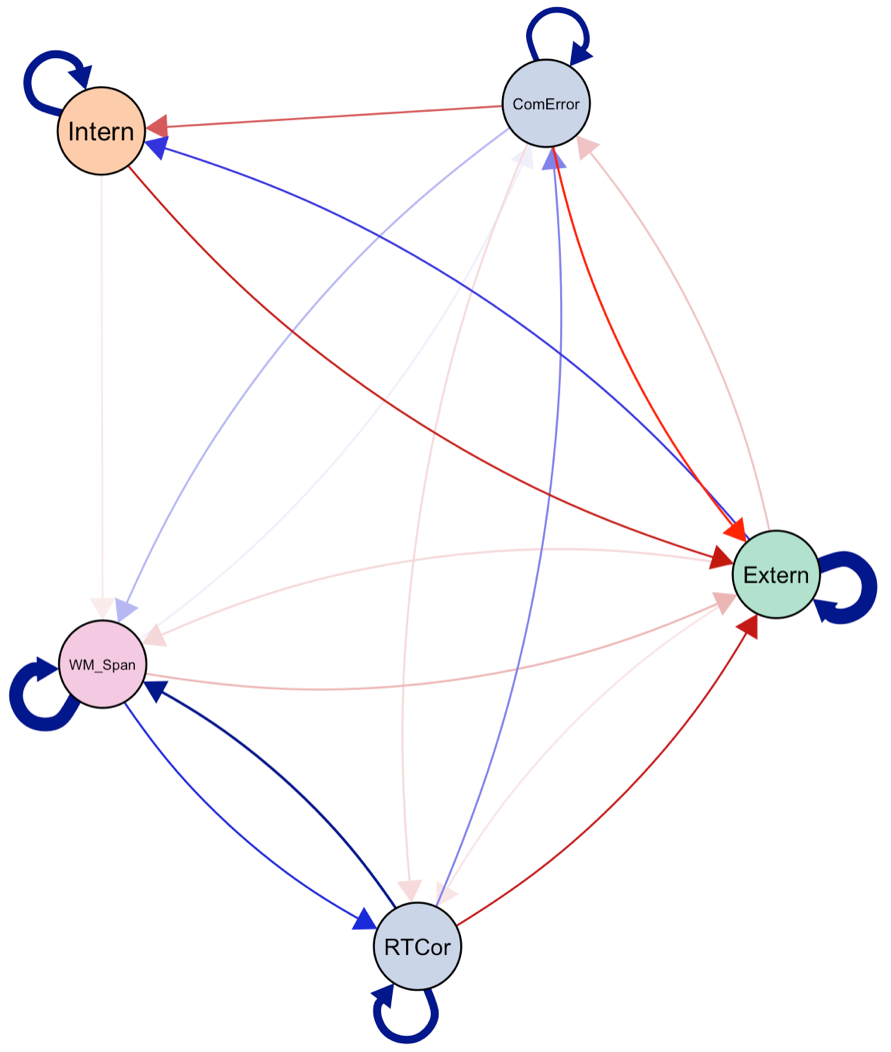

Supplement: Freichel et al. supplementary material [file S0033291725100639sup001.docx]
